# Association of famine exposure and the serum calcium level in healthy Chinese adults

**DOI:** 10.3389/fendo.2022.937380

**Published:** 2022-08-09

**Authors:** Yu-ying Yang, Deng Zhang, Ling-ying Ma, Yan-fang Hou, Yu-fang Bi, Yu Xu, Min Xu, Hong-yan Zhao, Li-hao Sun, Bei Tao, Jian-min Liu

**Affiliations:** ^1^ Department of Endocrine and Metabolic Diseases, Shanghai Institute of Endocrine and Metabolic Diseases, Ruijin Hospital, Shanghai Jiao Tong University School of Medicine, Shanghai, China; ^2^ Shanghai National Clinical Research Center for Metabolic Diseases, Key Laboratory for Endocrine and Metabolic Diseases of the National Health Commission of the PR China, Shanghai National Center for Translational Medicine, Ruijin Hospital, Shanghai Jiao Tong University School of Medicine, Shanghai, China

**Keywords:** famine, serum calcium, albumin-adjusted calcium, reference interval, hypercalcemia

## Abstract

**Objective:**

Famine exposure and higher serum calcium levels are related with increased risk of many disorders, including Alzheimer’s disease, atherosclerosis, diabetes, and osteoporosis. Whether famine exposure has any effect on serum calcium level is unclear. Besides, the normal reference range of serum calcium is variable among different populations. Our aims are 1) determining the reference interval of calcium in Chinese adults; 2) exploring its relationship with famine exposure.

**Methods:**

Data in this study was from a cross-sectional study of the epidemiologic investigation carried out during March-August 2010 in Jiading district, Shanghai, China. Nine thousand and two hundred eleven participants with estimated glomerular filtration rate (eGFR) ≥60ml/min/1.73m^2^ were involved to calculate reference interval of total calcium from 10569 participants aged 40 years or older. The analysis of famine exposure was conducted in 9315 participants with complete serum biochemical data and birth year information.

**Results:**

After rejecting outliers, the 95% reference interval of total serum calcium was 2.122~2.518 mmol/L. The equation of albumin-adjusted calcium was: Total calcium + 0.019* (49-Albumin), with a 95% reference interval of 2.151~2.500 mmol/L. Compared to the age-balanced control group, there was an increased risk of being at the upper quartile of total serum calcium (OR=1.350, 95%CI=1.199-1.521) and albumin-adjusted calcium (OR=1.381, 95%CI=1.234-1.544) in subjects experienced famine exposure in childhood. Females were more vulnerable to this impact (OR= 1.621, 95%CI= 1.396-1.883 for total serum calcium; OR=1.722, 95%CI= 1.497-1.980 for albumin-adjusted calcium).

**Conclusions:**

Famine exposure is an important environmental factor associated with the changes in circulating calcium concentrations, the newly established serum calcium normal range and albumin-adjusted calcium equation, together with the history of childhood famine exposure, might be useful in identifying subjects with abnormal calcium homeostasis and related diseases, especially in females.

## Introduction

Famine experience, especially during early life, has gathered increasing attention worldwide. Studies from the Dutch famine as well as the Great China’s Famine showed that experiencing food shortage during early life is associated with a higher risk of osteoporosis, vertebral fracture, type 2 diabetes, obesity, coronary artery disease, cognition decline, and schizophrenia (1–10). However, the underlying mechanism, especially the common causes or factors responsible for or related to these varieties of diseases, is poorly understood.

During the past two decades, mounts of evidence have revealed the interaction between skeleton metabolism and the functionalities of organs and systems (11–14). It is reported that bone resorption, with its consequence of motivating skeletal calcium into circulation, is one of the major mediating factors for such a connection (15–17). In fact, among those diseases related to famine exposure, many of them are also associated with higher serum calcium levels (18–23). For example, individuals having higher serum calcium, although still in the normal range, are at higher risk of intracranial atherosclerosis (18) and presence of calcified coronary atherosclerotic plaque (24), cognition decline, and clinical progression of Alzheimer’s disease (20), prevalence of adult overweight or obesity (25), incident type 2 diabetes (21), and lower bone mineral densities (BMDs) (22, 23). These studies indicated that elevated serum calcium level is not only an indicator but also the causal factor of the pathological processes. Thus, it makes establishing an adequate reference interval of serum calcium and finding its influencing factors essential to distinguish these pathological conditions at an early stage.

Serum calcium is closely regulated within an exquisitely narrow range. However, variation exists among different ethnics. A study regarding US civilian population showed that Mexican-Americans have lower serum calcium levels than Hispanics, while non-Hispanic blacks have higher serum calcium concentrations than non-Hispanic whites (26). On the other hand, in disease conditions like chronic kidney disease (CKD), black patients had lower serum calcium concentrations compared with white patients (27). Likely, the equation used to calculate albumin-adjusted calcium varies among different countries and regions (28, 29), and the use of population-specific equations improved the diagnostic accuracy of the adjusted calcium than the commonly used equation described by Payne et al. in 1973 (30, 31). These findings indicate that serum calcium levels may be influenced by ethnicity and it is necessary to determine adequate reference intervals as well as albumin-adjusted calcium equation regarding specific races, geographic regions and populations.

Serum calcium is regulated mainly by three systems: intestinal resorption, kidney reabsorption, and bone resorption. Low serum calcium or some pathological conditions can trigger a series of pathophysiological processes to increase calcium absorption by the intestines and reabsorption in the kidney (32, 33). More importantly, the release of calcium from the skeleton through bone resorption contributed significantly to an elevation of serum calcium level (34). In addition, recent studies reported that nutrition status may influence serum calcium level (35, 36) and famine exposure is also associated with metabolic bone abnormalities (7, 37). Thus, it is of interest and necessary to investigate whether serum calcium levels are affected by famine exposure.

In this study, we aimed to establish a reference interval of serum calcium level and equation for albumin-adjusted calcium in Chinese adults; and to explore the relationship between serum calcium level and famine exposure.

## Materials and methods

### Participants

**Figure 1:**
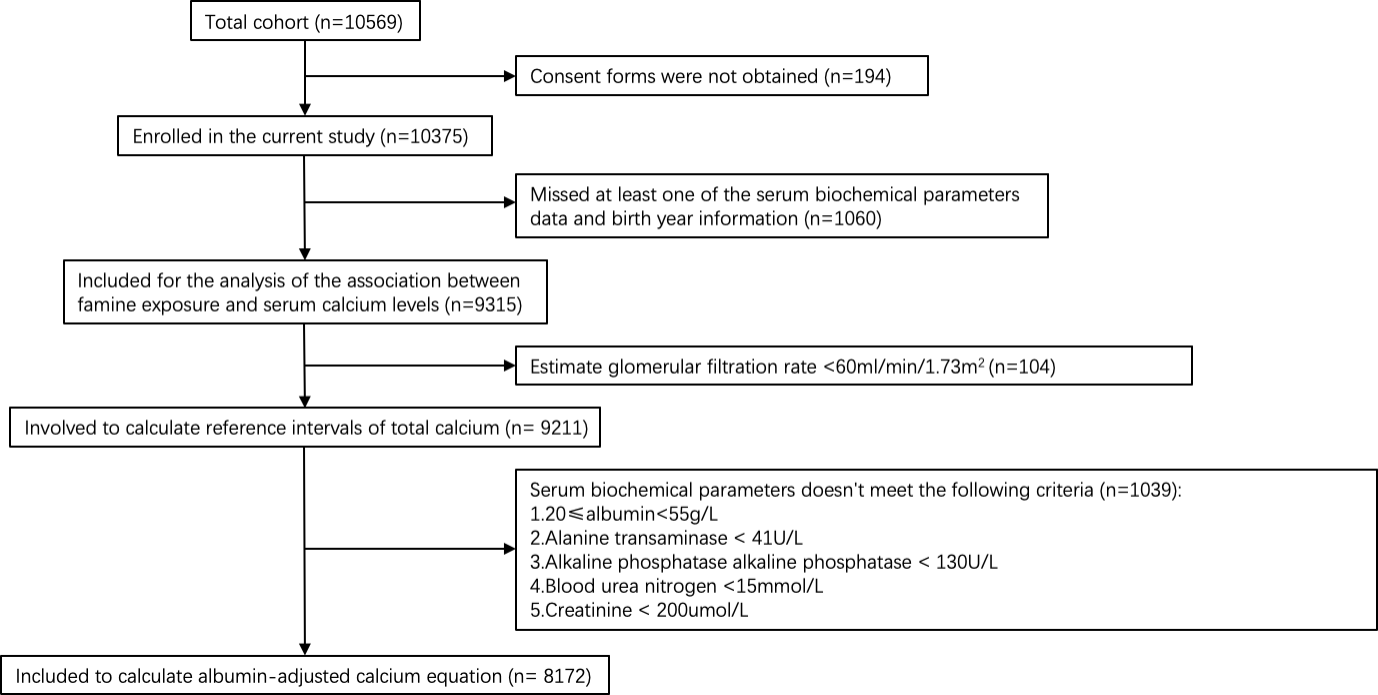
Flow chart of the participants selection of this study.

Data in this study were from a cross-sectional study of the epidemiologic investigation carried out during March-August 2010 in Jiading district, Shanghai, China (38). The study population was sampled using cluster sampling method. Ten thousand and five hundred sixty-nine men and women aged 40 years or older were invited by telephone or door-to-door visit to participate in this study. Among them, 10375 (98.2%) agreed to participate. 9211 participants with an estimated glomerular filtration rate (eGFR) ≥60ml/min/1.73m^2^ were involved to calculate reference intervals of total calcium. 8172 participants with normal hepatic and renal function(20≤albumin<55g/L, alanine transaminase (ALT)< 41U/L, alkaline phosphatase alkaline phosphatase (ALP) < 130U/L, blood urea nitrogen (BUN) <15mmol/L, creatinine (Cr) < 200umol/L) were included to calculate albumin-adjusted calcium equation according to the previously described protocol (39). And 9315 participants with complete serum biochemical parameters data and birth year information were included for the analysis of the association between famine exposure and serum calcium levels (**Figure 1**).

The study protocol was approved by the Institutional Review Board of the Rui-jin Hospital, Shanghai Jiao Tong University School of Medicine, and informed consent was obtained from all participants.

### Data collection

Anthropometric measurements were performed by the trained staff according to the standardized protocol. Height was measured to the nearest 0.1 cm, and weight was recorded to the nearest 0.1 kg with light clothing and no shoes. Body mass index (BMI) was calculated as body weight in kilograms divided by height squared in meters.

Venous blood samples were collected after an overnight fast. Serum Ca, P, ALT, AST, γGT, ALP, albumin, uric acid (UA), BUN, and Cr were measured using the autoanalyzer (Modular E170; Roche). CKD-EPI Creatinine Equation (2009) was used to calculate eGFR.

### Definition of famine exposure

The Great China’s Famine occurred from 1959 to 1962. According to the birth year, participants were divided into four groups: non-exposed (born after 1 Jan 1963), fetal exposure (born between 1 Jan 1959 to 31 Dec 1962), childhood exposure (born between 1 Jan 1949 to 31 Dec 1958), adolescent exposure (born between 1 Jan 1941 to 31 Dec 1948), and adulthood exposure (born before 31 Dec 1940).

Because there are no overlaps in the birth years among the 5 famine exposed groups, to limit the effect of age difference on the results, we combined the non-exposed (post-famine) group and adolescent exposure (pre-famine) group as the age-balanced control group of childhood exposure group (mean age: 55.94 vs 56.48 years) (40–42).

### Statistical analysis

To establish the total calcium reference interval, first, we excluded outliers that lay more than 3 quartiles above or below the interquartile range. The 95% reference interval was defined by mean ± 1.96*SD with 95% confidence intervals (95% CI).

The albumin-adjusted calcium equation was derived from the linear regression analysis using serum albumin as the dependent variable and serum calcium as the independent variable.

All continuous variables were presented as medians (interquartile ranges), and categorical variables were presented as proportions. Odds ratio (ORs) and 95% CIs were calculated using a multivariable-adjusted logistic regression model to analyze the association of famine exposure with serum total calcium and albumin-adjusted calcium. Mann–Whitney U-test was performed to compare serum total calcium levels and albumin-adjusted calcium between genders. A p-value less than 0.05 was considered statistically significant. The statistical analysis was performed using SPSS 23.0 (SPSS, Inc.)

## Results

### Reference interval for total calcium

Clinical characteristics of participants with consent forms and complete serum biochemical parameters were shown in **Table 1**. A total of 9211 participants with eGFR≥60ml/min/1.73m^2^ were involved to calculate reference intervals of total calcium. After rejecting outliers (n=1), the calculated results were shown in **Table 2**. The mean calcium level in the total cohort was 2.320mmol/L and females had higher mean total serum calcium levels than males (2.325 mmol/L vs 2.313 mmol/L, P=0.000).

**Table 1:** Clinical characteristics of participants according to famine exposure.

			Famine exposure			
	Non-exposed		Fetal	Childhood	Adolescent	Adult
**n**	1455	1097		3592	1911	1260
**Age (year)**	44.05 (42-46)	49.72 (49-51)		56.48 (54-59)	65.00 (63-67)	74.88 (72-77)
**Male (%)**	563 (38.69%)	408 (37.19%)		1253 (34.88%)	784 (41.03%)	553 (43.89%)
**BMI (kg/m2)**	24.53 (22.17-26.51)	25.02 (22.64-27.22)		25.20 (23.08-27.19)	25.39 (23.17-27.47)	24.93 (22.56-27.2)
**Ca (mmol/L)**	2.30 (2.24-2.36)	2.31 (2.25-2.38)		2.33 (2.27-2.4)	2.32 (2.25-2.38)	2.31 (2.25-2.38)
**ALB-adjusted Ca (mmol/L)**	2.30 (2.25-2.35)	2.32 (2.26-2.37)		2.33 (2.27-2.39)	2.33 (2.27-2.38)	2.34 (2.28-2.39)
**P (mmol/L)**	1.23 (1.09-1.36)	1.24 (1.09-1.39)		1.26 (1.13-1.4)	1.26 (1.11-1.41)	1.24 (1.11-1.38)
**ALB (g/L)**	48.90 (47.30-50.80)	48.86 (47.30-50.50)		49.02 (47.60-50.60)	48.53 (47.00-50.10)	47.52 (45.80-49.38)
**ALT (IU/L)**	23.65 (12.10-26.10)	21.97 (13.20-25.80)		22.55 (14.30-25.80)	21.76 (14.20-24.60)	19.59 (13.03-22.58)
**AST (IU/L)**	21.98 (16.30-23.20)	22.15 (17.30-23.95)		23.36 (18.50-25.40)	24.11 (19.10-26.30)	24.82 (19.63-27.20)
**γGT (IU/L)**	29.62 (13.00-31.00)	32.13 (14.00-35.00)		31.24 (15.00-33.00)	32.43 (16.00-36.00)	34.81 (16.00-38.00)
**ALP (IU/L)**	68.99 (56.00-79.00)	75.03 (60.00-86.00)		82.54 (67.00-95.00)	83.71 (69.00-96.00)	85.28 (68.00-99.00)
**UA (umol/L)**	279.82 (208.80-338.80)	286.17 (225.45-337.80)		298.60 (235.65-350.68)	311.23 (244.80-366.50)	322.89 (257.65-381.25)
**BUN (mmol/L)**	4.84 (4.00-5.50)	5.14 (4.20-5.90)		5.34 (4.40-6.10)	5.56 (4.60-6.30)	5.78 (4.60-6.60)
**Cr (umol/L)**	59.28 (49.40-67.50)	59.87 (50.60-66.90)		60.39 (51.40-67.40)	64.22 (53.50-71.10)	68.30 (56.03-77.20)
**eGFR (ml/min/1.73m2)**	110.29 (107.11-115.09)	105.52 (102.79-109.86)		99.64 (96.76-104.55)	91.71 (88.83-97.60)	82.70 (77.97-90.32)

Data were present as median (interquartile range) or proportions.

BMI, body mass index; ALB, albumin; ALT, alanine transaminase; AST, aspartate transaminase; γGT, Gamma Glutamyl Transpeptidase; ALP, alkaline phosphatase; UA, serum uric acid; BUN, blood urea nitrogen; Cr, serum creatin; eGFR, estimated glomerular filtration rate.

**Table 2:** Calculated reference intervals of serum total calcium and albumin-adjusted calcium.

	The lower limit of 95% reference interval (mmol/L) (95% confidence intervals)	The upper limit of 95% reference interval (mmol/L) (95% confidence intervals)
**Serum total calcium (mmol/L)**		
Total cohort	2.122 (2.120-2.124)	2.518 (2.516-2.520)
Male	2.113 (2.110-2.117)	2.512 (2.509-2.515)
Female	2.128 (2.125-2.130)	2.521 (2.519-2.524)
**Albumin-adjusted calcium (mmol/L)**		
Total cohort	2.151 (2.149-2.153)	2.500 (2.498-2.501)
Male	2.141 (2.138-2.144)	2.488 (2.485-2.491)
Female	2.159 (2.157-2.161)	2.505 (2.503-2.508)

### Albumin-adjusted calcium equation

**Figure 2:**
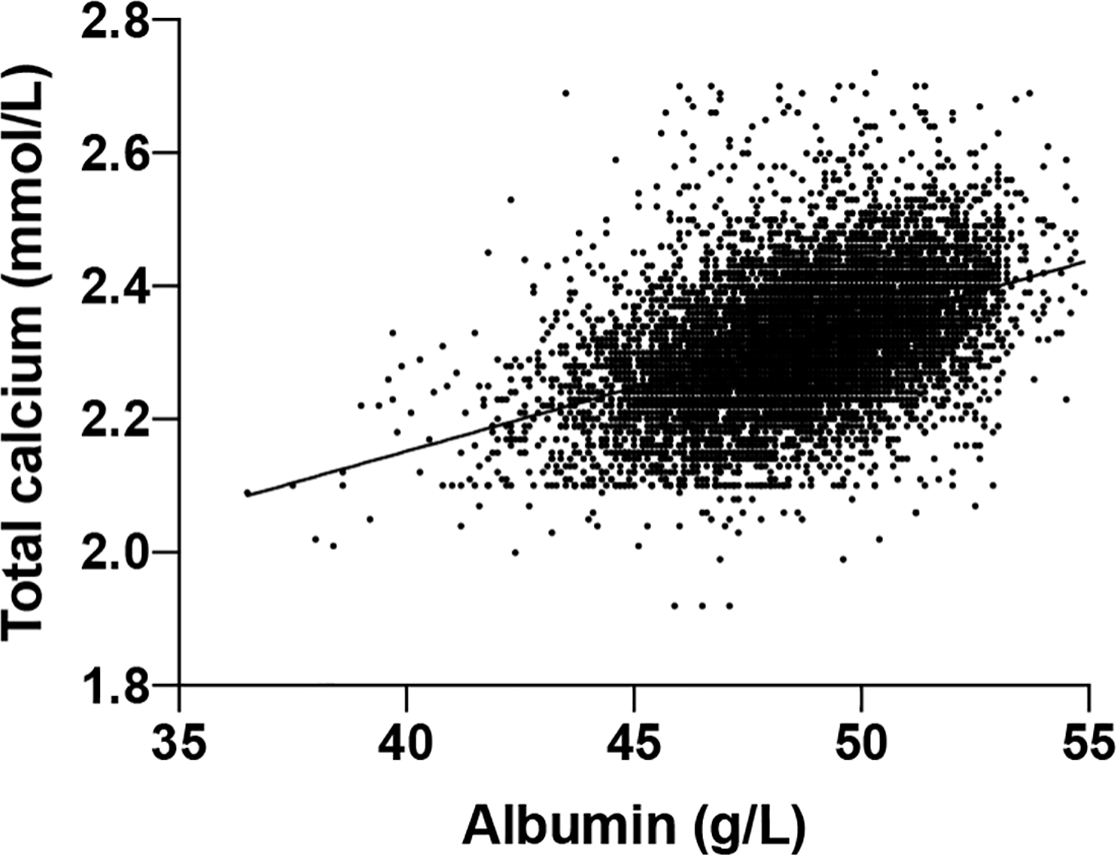
Scatter plot of total calcium (y-axis) and albumin (x-axis).

To derive the albumin-adjusted calcium, first, we established the albumin-adjusted calcium equation based on 8172 participants selected according to the previously protocol (39). The y-intercept was 1.389 and the slope was 0.019 (**Figure 2**) which made the equation:

Total calcium (mmol/L) = 0.019*albumin(g/L) + 1.389.

It is established that albumin-adjusted calcium=total calcium-(slope*albumin) + (mean normal total calcium-intercept calcium) (39), which is:

Albumin-adjusted calcium = Total calcium + 0.019 × (albumin) (mean total calcium–1.389)

Albumin-adjusted calcium = Total calcium + 0.019 × (albumin) (2.320- 1.389)

Albumin-adjusted calcium = Total calcium + 0.019 × (albumin) 0.931

Albumin-adjusted calcium = Total calcium + 0.019 × (49-Albumin)

### Reference interval for albumin-adjusted calcium

We calculated the albumin-adjusted calcium according to the above equation for 9211 participants with eGFR≥60ml/min/1.73m^2^. After rejecting outliers (11 outliers, 0.12%) by the method mentioned above, 9200 participants were included for the calculation of the reference interval of albumin-adjusted calcium, and the results were shown in **Table 2**. The mean albumin-adjusted calcium level in the total cohort was 2.325mmol/L and females had higher mean albumin-adjusted serum calcium levels than males (2.332 mmol/L vs 2.314 mmol/L, P=0.000).

### Clinical characteristics of participants according to famine exposure

We further studied whether exposure to famine at different stages of life had any effect on the serum calcium level in adulthood. The characteristics of 9315 participants were listed according to different life stages of exposure to famine. As shown in **Table 1**, participants who experienced famine had higher total calcium levels as well as albumin-adjusted calcium levels compared to non-exposed participants. Those exposed in childhood had the highest total calcium level and those exposed in adulthood had the highest albumin-adjusted calcium level.

### The association between famine exposure and serum calcium levels

To further evaluate the association between famine exposure and calcium level. We assorted the participant according to the quartiles of total calcium and analyzed the relationship between famine exposure and being at the upper quartile of total calcium. The ORs (95% CI) of being at the upper quartile of total calcium levels were 1.28(1.06-1.55), 1.67(1.44-1.94), 1.25(1.05-1.48), and 1.27(1.05-1.52) for fetal, childhood, adolescent and adulthood exposure to famine, respectively. After adjusting for age, sex, eGFR, and albumin, only participants who experienced famine in childhood had a significantly higher risk (OR=1.87, 95%CI=1.31-2.67) of being at the upper quartile of serum calcium (**Table 3**).

**Table 3:** | ORs (95% CI) for being at the upper quartile of serum total calcium of exposure to famine at different life stages.

Famine exposure						
Non-exposed		Fetal	Childhood	Adolescent	Adult
Whole cohort					
Case/total	279/1455	256/1097	1021/3592	436/1911	291/1260
Model 1	1.00(ref)	**1.28(1.06-1.55)**	**1.67(1.44-1.94)**	**1.25(1.05-1.48)**	**1.27(1.05-1.52)**
Model 2	1.00(ref)	1.33(0.95-1.86)	**1.99(1.44-2.76)**	1.72(0.82-3.63)	**4.295(1.72-10.75)**
Model 3	1.00(ref)	1.21(0.84-1.74)	**1.87(1.31-2.67)**	1.30(0.59-2.88)	2.08(0.76-5.67)
**Male**					
Case/total	151/563	87/408	274/1253	153/784	108/553
Model 1	1.00(ref)	0.739(0.55-1.00)	**0.764(0.61-0.96)**	**0.662(0.51-0.86)**	**0.662(0.50-0.88)**
Model 2	1.00(ref)	0.879(0.54-1.44)	1.046(0.61-1.80)	1.068(0.33-3.41)	3.744(0.91-15.49)
Model 3	1.00(ref)	0.86 (0.50-1.46)	0.855(0.50-1.46)	0.855(0.25-2.97)	2.305(0.49-10.84)
**Female**					
Case/total	128/892	169/689	747/2339	283/1127	183/707
Model 1	1.00(ref)	**1.94(1.50-2.50)**	**2.801(2.28-3.44)**	**2.001(1.59-2.52)**	**2.085(1.62-2.68)**
Model 2	1.00(ref)	**1.854(1.17-2.95)**	**3.238(2.13-4.92)**	**2.991(1.12-8.02)**	**5.348(1.58-18.06)**
Model 3	1.00(ref)	1.623(0.99-2.67)	**2.915(1.85-4.60)**	1.974(0.69-5.62)	2.197(0.58-8.27)

Model 1: unadjusted.

Model 2: adjusted for age and sex.

Model 3: adjusted for age, sex, eGFR, and ALB.Bold values are ORs reached statistical significance.

It was reported that females are more vulnerable to famine-associated metabolic dysregulation (43–46). We wondered whether there was bias in the influence of famine exposure on serum calcium levels between males and females. As demonstrated in **Table 3**, after multivariable adjustment, female participants, not males, who experienced famine in childhood were more likely to have higher calcium levels. Similar results were derived in the analysis of famine exposure and being upper quartile of albumin-adjusted calcium. Female participants who experienced famine in both fetal and childhood were more likely to have higher albumin-adjusted calcium levels after multivariable adjustment (**Table 4**).

**Table 4:** ORs (95% CI) for being at the upper quartile of serum albumin-adjusted calcium of exposed to famine at different life stages.

Famine exposure					
Non-exposed		Fetal	Childhood	Adolescent	Adult
**Whole cohort**					
Case/total	234/1455	248/1097	984/3592	486/1911	376/1260
Model 1	1.00(ref)	**1.52(1.25-1.86)**	**1.97(1.68-2.31)**	**1.78(1.50-2.12)**	**2.22(1.85-2.67)**
Model 2	1.00(ref)	1.23(0.87-1.75)	**2.13(1.52-2.99)**	1.24(0.59-2.63)	1.99(0.84-4.73)
Model 3	1.00(ref)	1.24(0.87-1.76)	**2.15(1.53-3.02)**	1.28(0.60-2.72)	2.00(0.84-4.78)
**Male**					
Case/total	119/563	69/408	237/1253	161/784	137/553
Model 1	1.00(ref)	0.76(0.55-1.06)	0.87(0.68-1.11)	0.96(0.74-1.26)	1.23(0.93-1.63)
Model 2	1.00(ref)	0.61(0.35-1.03)	0.94(0.53-1.69)	0.68(0.21-2.27)	1.28(0.33-4.88)
Model 3	1.00(ref)	0.64(0.37-1.10)	0.98(0.55-1.75)	0.73(0.22-2.44)	1.20(0.31-4.62)
**Female**					
Case/total	115/892	179/689	747/2339	325/1127	239/707
Model 1	1.00(ref)	**2.37(1.83-3.07)**	**3.17(2.56-3.93)**	**2.74(2.17-3.46)**	**3.45(2.69-4.43)**
Model 2	1.00(ref)	**2.06(1.28-3.32)**	**3.59(2.35-5.49)**	2.17(0.82-5.75)	2.88(0.92-9.04)
Model 3	1.00(ref)	**2.05(1.27-3.30)**	**3.59(2.34-5.50)**	2.19(0.82-5.82)	3.11(0.98-9.89)

Model 1: unadjusted.

Model 2: adjusted for age and sex.

Model 3: adjusted for age, sex and eGFR.Bold values are ORs reached statistical significance.

To control the effect of age on the outcomes of famine, we compared the childhood exposure group with the age-balanced control group. The results showed that exposure to famine in childhood was related to higher total serum calcium (OR=1.350, 95%CI=1.199-1.521) and albumin-adjusted calcium (OR=1.381, 95%CI=1.234-1.544) in their adulthood. Subgroup analysis showed that this effect only existed in females (**Tables 5**, **6**).

**Table 5:** The risk of being at the upper quartile of serum total calcium in later life following exposure to famine during childhood using age-balanced control group.

	Age-balanced control	Childhood exposure
**Whole cohort**		
Case/total	715/3366	1021/3592
Model 1	1.00(ref)	**1.472(1.319-1.643)**
Model 2	1.00(ref)	**1.454(1.302-1.623)**
Model 3	1.00(ref)	**1.350(1.199-1.521)**
**Male**		
Case/total	304/1347	274/1253
Model 1	1.00(ref)	0.960(0.798-1.155)
Model 2	1.00(ref)	0.976(0.811-1.176)
Model 3	1.00(ref)	1.008(0.825-1.231)
**Female**		
Case/total	411/2019	747/2339
Model 1	1.00(ref)	**1.836(1.598-2.109)**
Model 2	1.00(ref)	**1.842(1.601-2.118)**
Model 3s	1.00(ref)	**1.621(1.396-1.883)**

Model 1: unadjusted.

Model 2: adjusted for age and sex.

Model 3: adjusted for age, sex, eGFR, and ALB.Bold values are ORs reached statistical significance.

**Table 6:** The risk of being at the upper quartile of serum albumin-adjusted calcium in later life following exposure to famine during childhood using age-balanced control group.

	Age-balanced control	Childhood exposure
**Whole cohort**		
**Case/total**	720/3366	984/3592
Model 1	1.00(ref)	**1.387(1.242-1.548)**
Model 2	1.00(ref)	**1.367(1.223-1.527)**
Model 3	1.00(ref)	**1.381(1.234-1.544)**
**Male**		
Case/total	280/1347	237/1253
Model 1	1.00(ref)	0.889(0.733-1.078)
Model 2	1.00(ref)	0.890(0.733-1.079)
Model 3	1.00(ref)	0.912(0.751-1.108)
**Female**		
Case/total	440/2019	747/2339
Model 1	1.00(ref)	**1.684(1.469-1.931)**
Model 2	1.00(ref)	**1.718(1.495-1.974)**
Model 3	1.00(ref)	**1.722(1.497-1.980)**

Model 1: unadjusted.

Model 2: adjusted for age and sex.

Model 3: adjusted for age, sex and eGFR.Bold values are ORs reached statistical significance.

## Discussion

In this study, we provided the reference interval of serum calcium and established a new albumin-adjusted calcium equation for Chinese adults, and further found that participants who experienced famine in early life (fetal and childhood) have higher total and albumin-adjusted serum calcium levels, especially in females.

In a most recent study, more than 170 thousand European residents were investigated to derive reference interval of serum calcium (29). In our study, we also used a large population cohort to derive the Chinses-specific reference intervals of both total and albumin-adjusted serum calcium. The intervals were relatively narrow in our cohort, indicating that the cohort we used had relatively adequate homogeneity and representativeness. In addition, due to the variation among populations and methodology used for the measurement of total calcium and albumin levels, the use of a locally derived albumin-adjusted calcium equation is recommended by the Association for Clinical Biochemistry and Laboratory Medicine (ACB) (28). Our study provided a new equation for calculating albumin-adjusted calcium, which derived a higher value than commonly used equation (adjusted-calcium(mmol/L) = total calcium(mmol/L) + 0.02(40-albumin)) and the equation reported by the European study (adjusted-calcium(mmol/L) = total calcium(mmol/L) + 0.0177(45.2-albumin)) (29). It is noteworthy that compared to the European study, the average total and albumin-adjusted calcium concentrations were lower in our cohort. It is reported that the corrected serum calcium level of African-Americans is higher than Caucasians and Hispanics (47). More evidence is needed to confirm that the difference of circulating calcium between Asians and Europeans is caused by race.

In subgroup analysis, we found that the mean total and albumin-adjusted calcium concentrations were higher in females, which is in line with the European study. The reason behind such a phenomenon is multi-faceted: females go through a rapid estrogen decline during menopause, which augments the bone resorption rate. Thus, postmenopausal women have higher serum calcium than premenopausal women (48). In the meantime, males undergo a relatively moderate transition and serum calcium falls with aging, thus, in the aged population, females have higher serum calcium level than males (48). In our study, almost 80% of the participants were over 50 years old and this may be responsible for the higher serum calcium level in females of the total cohort. When we analyzed the participants under 50 years old, the results showed that serum calcium level was higher in males (data not shown), which is also in line with the previous reports (48, 49). All these findings suggest that it might be necessary to use country-specific or ethnic specific and even gender-specific reference intervals of serum calcium as well as albumin-adjusted calcium equation.

Despite serum calcium concentration being strictly regulated within an exquisitely narrow range, our study found that it is influenced by famine exposure during early life. Malnutrition is a predominant result caused by edible food deprivation during famine exposure (50). A study reported that thin children (16% of BMI lower than normal control) have a higher level of bone resorption marker C-terminal telopeptide of collagen type I (CTX) than normal-weight peers (51). Similar results are found in anorexia nervosa (AN) patients. In AN, although serum calcium concentration remains in the normal range, urinary calcium excretion is elevated while intestinal calcium absorption is unchanged (35, 36), indicating the loss of calcium from the skeleton due to increased bone resorption. Thus, increased serum calcium in individuals exposed to famine may be caused by enhanced bone resorption triggered by a nutrition deficiency.

Measuring serum total as well as albumin-adjusted calcium level and establishing their normal reference range is not only indispensable for diagnosing diseases with overt disturbed calcium metabolism such as hyperparathyroidism and hypoparathyroidism but its variations, even within the normal range, are also associated with other extra-skeleton disorders. It was reported that subjects in the upper three quartiles of corrected serum calcium concentration had a significantly increased risk for intracranial atherosclerosis compared with the lowest quartile (18). The prevalence of overweight/obesity almost doubled in the upper total serum calcium level quartile compared to the lowest quartile (25). In two longitudinal studies, participants who had higher serum calcium were 1.6-2.3 times risky to develop diabetes and AD during 2-8 years of follow-up (20, 21). More importantly, the causal effect of high calcium level on lower BMDs at lumber-spine and whole-body is confirmed by two recent Mendelian-randomization (MR) studies, this effect is even independent of the most three important calcium-modulating hormones: parathyroid hormone (PTH), vitamin D, and phosphate concentrations. Similarly, clinical trials demonstrated that high-dose (10000IU daily) vitamin D supplementation (with 9% of study participants experiencing hypercalcemia at the end of the trial) leads to accelerated bone loss compared to low-dose(400IU daily and none of hypercalcemia) (52, 53). The results derived from our study that early-life famine exposure has an impact on serum calcium concentrations in adulthood emphasized that for those with famine exposure in early life, it is necessary to evaluate their serum calcium level. This might be important to screen subjects at high risk of the above-mentioned calcium-related and famine-related diseases. In addition, whether there is a need to re-evaluate the reference interval of serum calcium and albumin-adjusted calcium equation when the nutrition status is greatly changed (improved) in the future is another interesting topic.

In subgroup analysis, we found that after multivariable adjustment, the relationship between famine and serum calcium only exists in female participants. One explanation is that during evolution, mammalian females have been exposed to more severe selection pressure than males during food shortages (54). Besides, in Chinese traditional culture, parents were intended to provide better nutrition to boys than girls when facing food shortages (55), thus there might be a severity difference in famine exposure between females and males.

The mechanism underlying elevated serum calcium and famine in early life is unclear; it might be related to enhanced bone resorption during famine exposure. In rodents, food restriction reduces cortical bone mass and cortical thickness while trabecular percent bone volume (BV/TV) was significantly lower in the food restriction group (56–58). Moreover, there is an increase in osteoclasts number and bone resorption in caloric restriction mice (59, 60), which is in line with the previous hypothesis that bone resorption activity was enhanced during famine exposure. Further studies revealed that serum leptin, which inhibits osteoclast generation (61), is decreased in food restriction mice (57, 59, 60), which may lead to the activation of osteoclastogenesis. On the other hand, dietary energy restriction elevates glucocorticoid hormone levels (62), and methylprednisolone treatment will increase osteoclast activity (63). However, more evidence is needed to support the hypothesis that famine exposure may result in increased bone resorptive activity and thus higher serum calcium levels.

There are some limitations in this study. Firstly, serum concentrations of PTH and 25OHD were not measured, which have critical roles in maintaining calcium homeostasis. However, vitamin D metabolism has been shown to behave normally in malnourished children (64) and serum calcium doesn’t relate to PTH levels in AN patients (35). It indicates that there are other mechanisms to regulate serum calcium in undernutrition conditions. Secondly, the serum concentrations of bone resorption and bone formation markers were not evaluated in our study. Thirdly, the severity and precise duration of famine exposure, confounding including place of birth and residence, and familial socioeconomic status (SES) at the time of the famine are unknown, thus the potential dose-response relationship between famine and serum calcium has not been studied in our research. A recent review addressed some recommendations that might help improve future Chinese famine studies (65). Besides, further studies regarding the mediation effect of calcium in famine-related health outcomes are needed.

## Conclusion

Our results suggest famine exposure is an important environmental factor responsible for the changes in circulating calcium concentrations, the newly established normal range of serum calcium and albumin adjusted calcium equation, together with the history of famine exposure in childhood, might be helpful in early identifying subjects with abnormal calcium homeostasis and related diseases, especially in females.

## Data availability statement

The raw data supporting the conclusions of this article will be made available by the authors, without undue reservation.

## Ethics statement

The studies involving human participants were reviewed and approved by Institutional Review Board of the Rui-jin Hospital, Shanghai Jiao Tong University School of Medicine. The patients/participants provided their written informed consent to participate in this study.

## Author contributions

J-mL, L-hS, and BT designed the study, Y-yY analyzed the data and write the manuscript. DZ and Y-fH verified the underlying data reported in the manuscript. L-yM, Y-fB, YX, and MX collect the data. J-mL interpreted the results and revised the manuscript. All authors contributed to the article and approved the submitted version.

## Acknowledgments

This research did not recieve any specific grant form any funding agency in the public, commercial or not-for-frofit sector.

## Conflict of interest

The authors declare that the research was conducted in the absence of any commercial or financial relationships that could be construed as a potential conflict of interest.

The reviewer HY declared a shared affiliation with the author(s) to the handling editor at the time of review.

## Publisher’s note

All claims expressed in this article are solely those of the authors and do not necessarily represent those of their affiliated organizations, or those of the publisher, the editors and the reviewers. Any product that may be evaluated in this article, or claim that may be made by its manufacturer, is not guaranteed or endorsed by the publisher.
